# Identification of Characteristic Genomic Markers in Human Hepatoma HuH-7 and Huh7.5.1-8 Cell Lines

**DOI:** 10.3389/fgene.2020.546106

**Published:** 2020-10-09

**Authors:** Masaki Kawamoto, Toshiyuki Yamaji, Kyoko Saito, Yoshitaka Shirasago, Kazuhiro Satomura, Toshinori Endo, Masayoshi Fukasawa, Kentaro Hanada, Naoki Osada

**Affiliations:** ^1^Graduate School of Information Science and Technology, Hokkaido University, Sapporo, Japan; ^2^Department of Biochemistry & Cell Biology, National Institute of Infectious Diseases, Tokyo, Japan; ^3^Faculty of Information Science and Technology, Hokkaido University, Sapporo, Japan; ^4^Global Station for Big Data and Cybersecurity, Global Institution for Collaborative Research and Education (GI-CoRE), Hokkaido University, Sapporo, Japan

**Keywords:** Huh7 cell line, genome sequencing, cell lines, hepatitis C virus, cell substrate

## Abstract

The human hepatoma-derived HuH-7 cell line and its derivatives (Huh7.5 and Huh7.5.1) have been widely used as a convenient experimental substitute for primary hepatocytes. In particular, these cell lines represent host cells suitable for propagating the hepatitis C virus (HCV) *in vitro*. The Huh7.5.1-8 cell line, a subline of Huh7.5.1, can propagate HCV more efficiently than its parental cells. To provide genomic information for cells’ quality control, we performed whole-genome sequencing of HuH-7 and Huh7.5.1-8 and identified their characteristic genomic deletions, some of which are applicable to an in-house test for cell authentication. Among the genes related to HCV infection and replication, 53 genes were found to carry missense or loss-of-function mutations likely specific to the HuH-7 and/or Huh7.5.1-8. Eight genes, including *DDX58* (*RIG-I*), *BAX*, *EP300*, and *SPP1* (osteopontin), contained mutations observed only in Huh7.5.1-8 or mutations with higher frequency in Huh7.5.1-8. These mutations might be relevant to phenotypic differences between the two cell lines and may also serve as genetic markers to distinguish Huh7.5.1-8 cells from the ancestral HuH-7 cells.

## Introduction

Quality control of cells is crucial for biopharmaceutical manufacturing as well as research. In research, many cell lines have been misidentified^1^, and various academic journals have begun requesting proper authentication of cells used in studies. Cell lines can be identified by genotyping multiple common genetic variants, such as microsatellite markers and single nucleotide variants (SNVs) (Gilbert et al., 1990). However, such common markers would not be useful for discriminating between cell lines that originated from the same ancestral cell line. Thus, rational, easy in-house methods of identifying cell lines are widely desired. In particular, the mutation rate of deletion is much lower than that of point mutations (Wang et al., 2008); cell line-specific deletions are applicable to cell line authentication of African green monkey kidney-derived Vero cells (Osada et al., 2014; Sakuma et al., 2018), which are widely used in virologic research and human vaccine production.

HuH-7 (hereafter Huh7) is a permanent cell line established from male hepatoma tissue, which was surgically removed from a 57-year-old Japanese male in 1982 (Nakabayashi et al., 1982). Huh7 and its derivatives have been used as a convenient experimental substitute for primary hepatocytes. Approximately 80% of hepatocellular carcinoma incidents in humans are caused by hepatitis viruses; in all hepatocellular carcinoma incidents, ∼25% were caused by hepatitis C virus (HCV) and ∼53% were caused by hepatitis B virus (HBV) (Perz et al., 2006). HCV is a positive-stranded RNA virus that infects humans, causing acute and chronic liver diseases. The chronic infection eventually leads to severe symptoms, such as hepatitis and hepatocellular carcinoma. Thus, permanent cell lines suitable for investigating human hepatitis viruses have always been invaluable. After the establishment of Huh7, several studies have attempted to derive cell lines that are more permissive to HCV than Huh7 (Blight et al., 2002; Feigelstock et al., 2010). Huh7.5, a subline of Huh7, was established as a highly permissive cell line to replicate subgenomic and genomic HCV RNA in 2002 (Blight et al., 2002). Although cell culture systems that recapitulate the HCV life cycle have not been developed for a long time, the JFH-1 HCV strain has been found to produce infective progenitor virions after introduction to Huh7.5 cells (Wakita et al., 2005). Interestingly, Huh7.5, but not the ancestral Huh7, has a missense mutation in *DDX58* (or *RIG-I*) gene, which participates in intracellular antiviral defense (Sumpter et al., 2005). Huh7.5.1, a subline of Huh7.5 cells, was subsequently established as a cell line with the intent to generate a cell line that cultures JFH-1 more efficiently than Huh7.5 (Zhong et al., 2005). Although Huh7.5.1 displayed faster viral replication kinetics than Huh7, Huh7, and Huh7.5.1 eventually produced a similar level of viral titers (Zhong et al., 2005). We previously found that the expression level of CD81, which is a plasma membrane protein essential for HCV infection (Petracca et al., 2000), was not uniform in the Huh7.5.1 cells, and we thus obtained Huh7.5.1-8, a subclone of Huh7.5.1, in which CD81 is stably expressed on cell surfaces (Shirasago et al., 2015). Huh7.5.1-8 cells exhibit ∼10-fold greater permissiveness to HCV than Huh7.5.1 cells (Shirasago et al., 2015). HCV culture systems have provided robust assay systems to screen anti-HCV compounds, resulting in the development of various anti-HCV medicines that are currently marketed, although anti-HCV vaccines have not been developed.

In this study, we determined the whole-genome sequences of Huh7 and Huh7.5.1-8 cell lines and found characteristic genomic deletions in Huh7 and Huh7.5.1-8, some of which are applicable to an in-house test for cell authentication. In addition, among the genes related to HCV infection and replication, 53 genes were found to carry missense or loss-of-function (LoF) mutations that were not registered in the public germline mutation databases, but were found in the Huh7 and/or Huh7.5.1-8. Among them, eight genes, including *DDX58* (*RIG-I*), *BAX*, *EP300*, and *SPP1* (osteopontin), contained mutations observed only in Huh7.5.1-8 or mutations with higher frequency in Huh7.5.1-8.

## Materials and Methods

### DNA Sample Preparation and Sequencing

Huh7 hepatocarcinoma cell line (JCRB0403) were obtained from the Japanese Collection of Research Bioresources (JCRB) Cell Bank. Huh7.5.1-8 cells were established as described previously (Shirasago et al., 2015). The HCV susceptibility is less in JCRB0403 than in Huh7.5.1-8 (Supplementary Figure 1), in line with previous studies (Murayama et al., 2012; Shirasago et al., 2015). For both samples, fragment libraries (average fragment sizes of ∼560 bp) were constructed using TruSeq DNA PCR-Free LT Library Prep Kit (Illumina, San Diego, CA, United States). Paired-end sequences of 150 bp long were determined using HiSeq X (Illumina). Approximately 1.8 billion sequence reads were obtained from each sample. We examined whether viral sequences of HBV and HCV are integrated into the two genomes using VirusFinder 2 software (Wang et al., 2015).

### RNA-Seq Data

We retrieved previously obtained RNA-seq data from Huh7.5.1 and Huh7.5.1-8 (DRR018792 and DRR018793, respectively). Because the initial quality check for the RNA-seq data showed some of the read bases had relatively low quality, we applied Trimmomatic software (version 0.36) (Bolger et al., 2014) to filter out low-quality reads. After trimming low-quality bases of average quality score <20 (window size: 4), reads shorter than 75 bp were filtered out. RNA-seq reads were mapped to the reference human genome (GRCh38) using HISAT2 (Kim et al., 2015).

### SNV Calling

Genomic reads were mapped to the reference human genome sequence (GRCh38 primary assembly downloaded from the Ensembl database) using the BWA MEM algorithm (version 0.7.15-r1140) (Li and Durbin, 2009) with a default parameter setting. The read mapping rates of Huh7 and Huh7.5.1-8 were 99.88 and 99.87%, respectively. To generate alignment files for variant calling, GATK Best Practices Pipeline 3.0, which includes duplicated read filtering, realignment around indels, and recalibration of base quality score, was applied to the initial alignments (McKenna et al., 2010).

The sites with read depths of ≥14 and ≤100 were used for the following variant calling. The number of sites with ≥14 read depth covered >95% of the reference human genome in our dataset. Initial SNV calling was performed using VarScan (version 2.4.3) (Koboldt et al., 2012) with a base quality score cutoff of 15 and a variant allele frequency cutoff of 0.1%. Multiallelic SNVs were removed from the analytical pipeline. Known germline SNVs registered in the public database (dbSNP_149) were filtered out using the SelectVariants program in the GATK package (McKenna et al., 2010). However, when a variant nucleotide in Huh7 and/or Huh7.5.1-8 was different from that in the dbSNP, those variants were kept for further analyses as a novel variant.

We defined three categories of newly identified SNVs according to their frequency in Huh7 and Huh7.5.1-8. We tested whether the SNV frequency is higher in Huh7 or Huh7.5.1-8 with statistical significance. Statistical significance was evaluated using the χ^2^ test and a false discovery rate of 0.05 (Benjamini and Hochberg, 1995). The SNVs with higher frequency in Huh7 and Huh7.5.1-8 were categorized into “Huh7-predominant” and “Huh7.5.1-8-predominant” categories, respectively. The SNVs that did not pass the criteria were further classified into “shared” SNVs when the variant frequencies in both samples exceeded 0.25.

### Structural Variant Calling

Indels shorter than 50 bp were identified using VarScan, with the same pipeline for SNV calling. Indels that exactly matched to known germline indels [Mills and 1000G gold standard indels (Mills et al., 2006)] were filtered out from further analyses. Accordingly, short indels were classified into three categories: Huh7-predominant, Huh7.5.1-8-predominant, and shared short indels.

Long indels (≥50 bp) were identified using Manta (version 1.1.0) (Chen et al., 2016). As with the SNV calling, we only considered the sites with read depths of ≥14 and ≤100. The estimated structural variant frequency in Manta was difficult when we did not obtain a sufficient number of reads spanning breakpoints. Therefore, we classified the structural variants into the above three categories without considering variant frequencies. Large insertions that match to the insertions in Mills and 1000G gold standard dataset and large deletions that overlapped (≥50% length of the estimated deletion size in the cell lines) with the deletions of Mills and 1000G gold standard and/or 1000 Genome Phase 3 structural variants (downloaded from ftp://ftp.1000genomes.ebi.ac.uk/vol1/ftp/phase3/) were filtered out.

### Functional Annotation

Functional annotation of mutations was performed using the snpEff software (Cingolani et al., 2012) with an annotation data snpEff_v4_3_GRCh38.86. Using the annotation information, SNVs with strong phenotypic effects (missense mutations, nonsense mutations, and mutations at splicing acceptor-donor sites) were extracted. Functional annotation of genes was performed using the DAVID bioinformatics resource (Jiao et al., 2012). We used the following five fields in DAVID output: disease, functional categories, gene ontology, pathways, and protein domains. The protein-protein association networks were retrieved from the STRING database (Szklarczyk et al., 2015). The Python scripts used in this study were deposited in GitHub^2^.

### Sequence Conservation

We investigated the evolutionary conservation at mutated amino acid sites using the amino acid sequences of 12 non-human vertebrate species: *Pan troglodytes*, *Pan paniscus*, *Gorilla gorilla, Pongo abelii*, *Macaca mulatta*, *Cricetulus griseus*, *Felis catus*, *Desmodus rotundus*, *Phascolarctos cinereus*, and *Gallus gallus.* Sequence data were retrieved and aligned using MEGAX (Knyaz et al., 2018), and sequence alignment was performed with the MUSCLE algorithm (Edgar, 2004).

### Experimental Validation of Deletions

The HeLa cervical carcinoma cell line (JCRB9004) was obtained from the JCRB Cell Bank and used as a control. Huh7.5 cells (Blight et al., 2002) were kindly provided from Dr. Charles M. Rice. Huh7.5.1 cells (Zhong et al., 2005) were kindly provided from Dr. Francis V. Chisari. Genomic DNA was prepared from the cell lines using the Blood Genomic DNA Extraction Mini Kit (Favorgen, Ping-Tung, Taiwan, ROC). Deletions in the genomes were verified by PCR experiments using a previously reported procedure (Sakuma et al., 2018). Briefly, PrimeSTAR GXL DNA Polymerase (Takara Bio, Otsu, Japan) was used for amplification. The reaction mixture, containing 60 ng genome DNA, was denatured at 98°C for 1 min and then subjected to 40 cycles, consisting of 98°C for 10 s, 61°C for 15 s, and 68°C, for 1 min. The amplified products were electrophoresed on an agarose gel and visualized with the Gel Doc EZ imager (Bio-Rad, Hercules, CA, United States). Then, 1 kb Plus DNA Ladder was used as a molecular marker (Thermo Fisher Scientific, Waltham, MA, United States).

### Sanger Sequencing of DDX58 Gene

Genomic DNA was prepared from 1 × 10^6^ cells using the Blood Genomic DNA Extraction Mini Kit (Favorgen) and used as a template to amplify a 446 bp fragment corresponding to bases 30,196–30,641 of the human DDX58 genic region (NCBI accession number NG_046918.1; 78,023 bp) using PCR. PCR was performed with 35 cycles of amplification (95°C for 10 s, 57°C for 5 s, and 72°C for 5 s) on the PCR Thermal Cycler Dice (Takara Bio) using PrimeSTAR MAX DNA polymerase (Takara Bio), forward primer 5′-GTGGCTTGGTGAAGAATGGGCACAG-3′ (bases 30,196–30,220), and reverse primer 5′-CTCAGACTAAGAGGCATGAACTATAAGTGG-3′ (complementary to bases 30,612–30,641). The resulting fragments were separated in an agarose gel, purified using with a gel/PCR extraction kit (FastGene; Nippon Genetics, Tokyo, Japan), and directly sequenced by the Sanger method using forward primer 5′-CCCTATTTGGGAAGGTCTGGTGATC-3′ (bases 30,291–30,315) and reverse primer 5′-CACTTTTACAGTATTGTCAAGCAGC-3′ (complementary to bases 30,564–30,588) by Eurofins Genomics K.K. (Tokyo, Japan).

## Results and Discussion

### Identification of Mutations in Huh7 Lineages

We obtained 41.0- and 41.1-fold coverages of whole-genome sequence reads from Huh7 (JCRB0403) and Huh7.5.1-8 cells, respectively. No sequences related to HBV and HCV were detected in the genomes using the VirusFinder2 pipeline, as reported previously for HBV using *in situ* hybridization (Tay et al., 1990). To identify novel mutations, we filtered out mutations matched to the previously known germline SNVs and indels from further analyses (see Materials and Methods). These mutations were not found in currently available human mutation database, suggesting that most (even if not all) of these newly identified SNVs specifically occurred during the establishment of the Huh7 cell line and/or the development of the liver cancer in the patient, from which the cell line was established. For the following analysis, we further classified mutations into three categories: 1) Huh7-predominant mutations, of which frequency is higher in Huh7 than in Huh7.5.1-8, 2) Huh7.5.1-8-predominant mutations, of which mutation frequency is higher in Huh7.5.1-8 than in Huh7, and 3) shared mutations, of which frequency is almost equal between the two cell lines. The SNVs of higher frequency in Huh7 than in Huh7.5.1-8, with statistical significance (false discovery rate of 0.05), were classified as Huh7-predominant SNVs and vice versa. The SNVs that did not pass the criteria were further classified into shared SNVs when the variant frequencies in both samples exceeded 0.25. Table 1 summarizes the number of mutations, including SNVs, insertions, and deletions.

**TABLE 1:** Summary of newly identified variants in Huh7 and Huh7.5.1-8.

Mutation type	Huh7-predominant	Huh7.5.1-8-predominant	Shared
SNV	4,094	39,497	183,856
Insertion	911	933	27,206
Deletion	1,898	8,948	126,084

As expected, many new mutations had higher frequencies in Huh7.5.1-8 than in Huh7 (Huh7.5.1-8-predominant class), because Huh7.5.1-8 should have experienced a larger number of cell passages. However, Huh7 also had a non-negligible number of mutations that were not found in Huh7.5.1-8. The observation was, presumably, accounted for by the fact that the seed stock of Huh7 cells we sequenced was not a direct ancestral cell seed that was used to establish Huh7.5. In total, we identified 394,568 new mutations, including 227,447 SNVs and 29,050 insertions, and 136,930 deletions.

Most of the mutations were shared between the two cell lines. These shared mutations were most likely to have arisen before the establishment of the Huh7 cell line. Heterogeneity of mutations is often seen not only among different cancer types but also among cancer cells of different patients diagnosed with the same cancer type. Indeed, the amount and pattern of new mutations in liver cancer tissues were shown to be considerably diverse (Fujimoto et al., 2015). In our study, Huh7 and Huh7.5.1-8 contained a relatively large number of new mutations, although it is impossible to obtain germline cells of the patient from whom Huh7 derived to sequence their genome, and some of the mutations might have occurred in the germline cells. Notably, the number of insertions and deletions was considerably high, and the ratio of indels to SNVs was much higher than the ratio observed in germline cells (Wang et al., 2008). Our validation, using polymerase chain reaction (PCR), confirmed that all six amplified regions contained specific deletions in the cell line DNA, indicating that the false-positive rate would not be substantially high in our variant calling pipeline.

Interestingly, we identified one non-synonymous mutation (K70R) in the *POLD3* gene, which plays an important role in high-fidelity DNA replication (Johansson and MacNeill, 2010) in both Huh7 and Huh7.5.1-8. In all 12 non-human vertebrates we analyzed, POLD3 had lysine residues at site 70, indicating that this amino acid site has a very important function in the protein. *POLD3* mutations have been identified repeatedly in cancer cells (Wang et al., 2014), and they contribute to chromosomal instability (Minocherhomji et al., 2015). In addition to *POL3D*, we found that an intron of the *XRCC4* gene, which encodes a DNA repair protein and plays a key role in the non-homologous end joining pathway (Yurchenko et al., 2006), had mutations at a splicing donner-acceptor site in both Huh7 and Huh7.5.1-8. We suspected that these mutations partly explain the large number of SNVs and indels and the frequent chromosome copy number changes in Huh7 (Kasai et al., 2018).

### Deletion Markers for Huh7 Cell Lines

To establish efficient deletion markers characteristic of Huh7 cells, we designed PCR primer sets that test the presence of deletion in genomic DNA using the dataset of homozygous deletions identified in whole-genome sequencing. Four long deletions, ranging from 7,400 to 262,700 bp in length, and four short deletions, ranging from 645 to 1,018 bp in length, were targeted for PCR amplification. We designated regions harboring long and short deletions as DL and DS regions, respectively. In our genome sequencing analysis, Five indels were expected to be present only in Huh7.5.1-8, whereas the others were expected to be shared between Huh7 and Huh7.5.1-8. We used a DNA sample from HeLa cells as a control. Primer sequences and detailed information for each region are presented in Supplementary Table 1.

**FIGURE 1:**
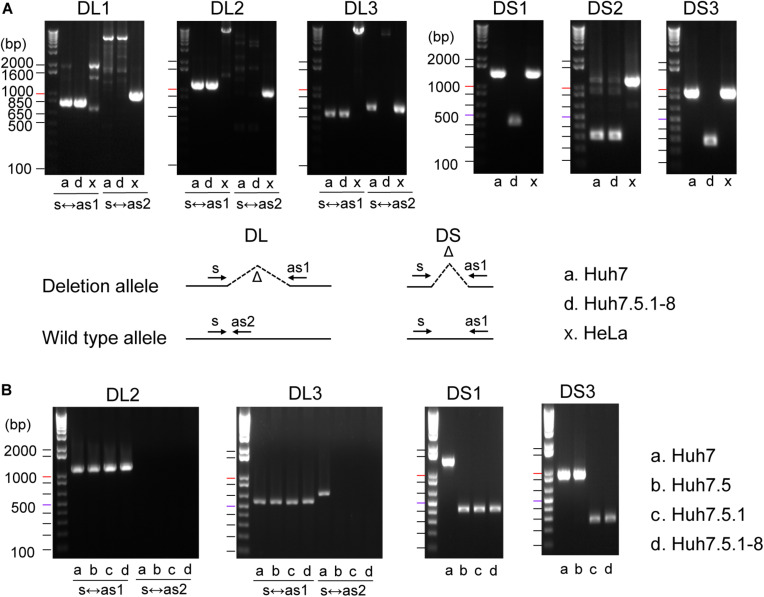
**(A)** Evaluation of deletions identified in Huh7 and Huh7.5.1-8 using PCR. Samples in lanes a, d, and x are DNA from Huh7, Huh7.5.1-8, and HeLa cells, respectively. Size markers are indicated by horizontal lines. The designs for PCR amplification and expected results are shown on the bottom of panel. The region name is given for each gel image. Prefixes DL and DS represent whether the expected deletion length is long (>7.4 kbp) or short (<0.9 kbp). For DL regions, primer pairs used for the experiments are shown below each image. For DS regions, primer pairs s and as1 were used for all regions. Each gel image was cropped from the full-sized gels, but different lanes were not merged into a single image. **(B)** Evaluation of deletions in Huh7, Huh7.5, Huh7.5.1, and Huh7.5.1-8 using PCR. Samples in lanes a, b, c, and d are DNA from Huh7, Huh7.5, Huh7.5.1, and Huh7.5.1-8, respectively.

We successfully amplified six regions by genomic PCR and confirmed that they all harbored deletions of expected sizes in Huh7 and/or Huh7.5.1-8, but did not show the signature deletion in the HeLa cells (Figure 1A). In total, four deletions (DL1–3 and DS2) were present both in Huh7 and Huh7.5.1-8, and two deletions (DS1 and DS3) were observed only in Huh7.5.1-8. Although the deletion in DL3 was present both in Huh7 and Huh7.5.1-8, Huh7 showed a PCR band corresponding to a non-deleted allele, indicating that the deletion was heterozygous in Huh7 but became homozygous in Huh7.5.1-8. The results are summarized in Supplementary Table 2.

The above experiment showed that three deletions (DL3, DL1, and DL3) could be useful to distinguish Huh7 and Huh7.5.1-8 cell lines. We further investigated whether these three characteristic deletions are present in the intermediate cell lines, Huh7.5, Huh7.5.1. Genomic PCR experiments verified that the deletion of DL2 exists in all Huh7, Huh7.5, Huh7.5.1, and Huh7.5.1-8 cell lines as expected (Figure 1B). Interestingly, the homozygous deletions at DL3 and DS1 were found to exist in Huh7.5, Huh7.5.1, and Huh7.5.1-8, but not in Huh7 while the deletion at DS3 was found to exist in Huh7.5.1 and Huh7.5.1-8, but not in Huh7 nor Huh7.5 (Figure 1B). Collectively, these results indicated, (1) DL2 can be used as a common genomic marker of Huh7-derived cell lines, (2) DS1 and DL3 (more accurately, homozygous existing of DL3) can be used to distinguish Huh7.5 and its derivatives from the parental Huh7 cell line, and (3) DS3 can be used to distinguish Huh7.5.1 and its derivatives from the parental Huh7.5 cell line. Of note, we failed to find deletions specific to Huh7.5.1-8 although it may be feasible to distinguish Huh7.5.1-8 cells from Huh7.5.1 cells by analyzing the stable and homogenous expression of CD81 on the cell surface (Murayama et al., 2012; Shirasago et al., 2015).

### Classification of Newly Identified SNVs

The newly identified SNVs were further classified into missense mutations, nonsense mutations, mutations at splicing signal sites, and other mutations. In addition, frameshift and large indels harboring protein-coding sequences (including gene fusions) were classified as LoF mutations. Nonsense mutations and mutations at splicing signal sites were also classified as LoF mutations.

Among these mutations, we selected genes that have missense and/or LoF mutations. We confirmed that missense mutations are present in the RNA-seq read data, which means that mutated alleles are actually expressed in the cells. In total, 255 and 431 genes were found to have missense and LoF mutations, respectively. Missense and LoF mutations were identified in genes on the X chromosomes but not on the Y chromosome. We also predict the effect of missense mutations using *in silico* prediction program, PROVEAN (Choi and Chan, 2015), using a cut-off score of -2.5. Among the 283 missense mutations, 81 were predicted as deleterious mutations, which potentially affect the phenotype of cell lines. The complete list of genes is shown in Supplementary Data 1.

We narrowed down the gene list and chose several genes that might be relevant to the HCV replication process. We reviewed previous research and selected keywords that are related to HCV infection and replication (Scheel and Rice, 2013; Bukh, 2016): autophagy, apoptosis, antiviral defense, hepatitis C, innate immune response, and serine protease. In addition, we surveyed the genes involved in protein-protein association networks with nine core genes that showed a strong influence on HCV infection and replication (*CD36*, *CD81*, *CLDN1*, *EGFR*, *EPHA2*, *LDLR*, *OCLN*, *PPIA*, and *SCARB1*).

We identified 53 candidate genes, including 12 autophagy-related genes, 22 apoptosis-related genes, 3 antiviral defense genes, 14 HCV-related genes, 8 innate immune-related genes, 4 serine proteases, and 4 genes involved in the HCV core-gene network. Eleven genes were categorized into two different classes. The list of genes and mutations is summarized in Table 2. Two HLA genes, *HLA-DRB1* and *HLA-DRB5*, related to an acquired immune system, contained several LoF mutations shared between Huh7 and Huh7.5.1-8, albeit those genes were not included in the 53 genes in Table 2 since cultured cells lack the acquired immune system. Interestingly, among the 53 candidate genes, 8 genes contained Huh7.5.8-1-predominant mutations, but none carried Huh7-predominant mutations. Among the 8 genes, 4 genes (*BAX*, *COL6A3*, *DEFB104B*, and *SIRPB1*) had LoF mutations, whereas the other 4 genes (*DDX58*, *EP300*, *SPP1*, and *ZNF654*) had missense mutations (Table 2). Future studies will be required to elucidate whether these mutations are relevant to the phenotypes of the Huh7 cell lineage.

**TABLE 2:** Mutations potentially contributing HCV permissiveness to Huh7 and Huh7.5.1-8.

Gene	Status	Type*	Ensembl transcript (AA type)	Function^†^
*BAX*	Huh7.5.1-8-predominant	CID	N/A	2
*COL6A3*	Huh7.5.1-8-predominant	SSM	N/A	7
*DEFB104B*	Huh7.5.1-8-predominant	CID	N/A	6
*SIRPB1*	Huh7.5.1-8-predominant	GFM	N/A	6
*ACIN1*	Shared	CID	N/A	2
*AR*	Shared	CID	N/A	5
*ATG3*	Shared	CID	N/A	1
*ATM*	Shared	SDA	N/A	1, 2
*ATP6AP2*	Shared	CID	N/A	2
*CD34*	Shared	CID	N/A	5
*CHMP4A*	Shared	CID	N/A	1, 2
*CTSA*	Shared	CID	N/A	1
*EGF*	Shared	CID	N/A	5, 4
*EIF2AK3*	Shared	CID	N/A	5
*EIF4G3*	Shared	CID	N/A	1
*GPX1*	Shared	CID	N/A	2
*H3CR1*	Shared	CID	N/A	5
*HTT*	Shared	CID	N/A	1, 2
*IFNA10*	Shared	SSM	N/A	3, 5, 6
*IFT20*	Shared	SDA	N/A	1
*KIR2DL4*	Shared	CID, SDA	N/A	5
*M6PR*	Shared	CID	N/A	4
*MAP3K1*	Shared	CID	N/A	2
*MMP17*	Shared	CID	N/A	5
*NOTCH4*	Shared	CID	N/A	5
*OVOS2*	Shared	CID	N/A	7
*PHLDA1*	Shared	CID	N/A	2
*PHLPP1*	Shared	CID	N/A	2
*PRUNE2*	Shared	CID	N/A	2
*PTEN*	Shared	SDA	N/A	2
*SNCA*	Shared	SDA	N/A	1
*TMBIM4*	Shared	GFM	N/A	2
*TNRC18*	Shared	CID, SSM	N/A	7
*TP53*	Shared	CID	N/A	5, 2, 4
*UBE2D3*	Shared	CID	N/A	2
*DDX58*	Huh7.5.1-8-predominant	Missense	ENST00000379883.2 (T55I)	3, 5, 6
*EP300*	Huh7.5.1-8-predominant	Missense	ENST00000263253.8 (D985G)	1
*EP300*	Huh7.5.1-8-predominant	Missense	ENST00000263253.8 (V1764I)	1
*SPP1*	Huh7.5.1-8-predominant	Missense	ENST00000508233.5 (K241T)	5
*ZNF654*	Huh7.5.1-8-predominant	Missense	ENST00000636215.1 (D1072N)	4
*ASAH2*	Shared	Missense	ENST00000489640.5 (C74Y)	2
*ATP6V0A2*	Shared	Missense	ENST00000613625.4 (G82E)	1, 7
*C4A*	Shared	Missense	ENST00000498271.1 (S347Y)	6
*CASP8AP2*	Shared	Missense	ENST00000551025.3 (K1245N)	2
*CYLD*	Shared	Missense	ENST00000427738.7 (T822N)	6
*DAB2IP*	Shared	Missense	ENST00000408936.7 (R1164C)	6, 2
*DIDO1*	Shared	Missense	ENST00000266070.8 (S1019C)	2
*DPF2*	Shared	Missense	ENST00000531989.1 (G41V)	2
*IL4R*	Shared	Missense	ENST00000563002.5 (A82T)	5
*PAK4*	Shared	Missense	ENST00000360442.7 (R175W)	2
*PIP4K2C*	Shared	Missense	ENST00000540759.6 (A300G)	1
*TBC1D25*	Shared	Missense	ENST00000376771.8 (N277S)	1
*TNFRSF10B*	Shared	Missense	ENST00000276431.8 (V191A)	5, 2
*TP53*	Shared	Missense	ENST00000509690.5 (Y88C)	5, 2, 4
*TRIM56*	Shared	Missense	ENST00000306085.10 (R395W)	3, 6

*Classes of LoF mutations: SDA, splice donor/acceptor mutation; GFM, gene fusion mutation; CID, coding sequence InDel; Missense, missense mutation; SSM, start/stop codon mutation. ^†^1, autophagy-related gene; 2, apoptosis-related gene; 3, antivirus-related gene; 4, genes involved in protein-protein association networks, with nine core genes that showed strong influence on HCV infection and replication; 5, known hepatitis C-related gene; 6, innate immune-related gene; 7, serine protease-related gene.

One of the genes, *DDX58*, which is also known as *RIG-I*, encodes an RNA helicase and has an important function for innate antiviral response (Yoneyama et al., 2004). The mutation is, therefore, a strong candidate for the high permissiveness of HCV in the Huh7.5 lineage. It is noteworthy that our whole-genome sequence approach also identified this gene. However, we found a difference in the mutation frequency between the results of this and previous studies. This incongruence is discussed in the next subsection.

Because we do not have sufficient space to discuss all 53 candidate genes for the higher permissiveness of HCV in detail, we here selected three key genes that have important viral replication functions.

BAX, which has an LoF mutation observed only in Huh7.5.1-8, forms a dimer with BCL2 and activates apoptosis (Oltval et al., 1993). In the Huh7 cell lineage, the *TP53* gene, which encodes P53 protein and controls the apoptotic process through BAX (Miyashita and Reed, 1995), has a frameshift deletion (Table 2). Therefore, BAX may positively regulate apoptosis in Huh7. In contrast, in Huh7.5.1-8, 22% of *BAX* alleles carried frameshift insertions, which would decrease *BAX*’s overall activity. The apoptotic activity, therefore, might have decreased in Huh7.5.1-8 compared with Huh7, which might result in HCV’s higher replication efficiency.

Another candidate gene, *EP300*, which encodes adenovirus early region 1A-associated protein p300 (Eckner et al., 1994; Ogryzko et al., 1996), harbored two missense mutations (D985G and V1764I) in Huh7.5.1-8. *EP300* has the function of suppressing the autophagy process (Pietrocola et al., 2014). Previous studies have shown that HCV and other flaviviruses replicate in host cells using autophagosomes (Levine et al., 2011; Fahmy and Labonté, 2017). We found that the valine residue at site 1764 was highly conserved among vertebrates. The mutation may have reduced *P300*’s function for autophagy suppression and contributed to efficient HCV replication.

The last gene we present here is *SPP1* (osteopontin), which is involved in the remodeling process of bones (Kahles et al., 2014). Previous studies found that (1) the gene expression of *SPP1* in liver tissue was increased according to the progress of hepatitis C (Asselah et al., 2005), (2) *SPP1* enhanced autophagy in human hepatocellular carcinoma cells (Liu et al., 2016), and (3) *SPP1* interacted with HCV proteins and helped replicate and assemble HCV (Iqbal et al., 2018). Indeed, it was reported that *SPP1* expression is up-regulated in Huh7.5 compared with Huh7 (Choi et al., 2014). Our study found that 46% of *SPP1* alleles in Huh7.5.1-8 carried missense mutations of K241T, whereas the frequency of mutation in Huh7 was 0%. The frequency of mutations in both Huh7.5.1 and Huh7.5.1-8 transcripts was close to 50%, indicating the K241T mutation had already been acquired in Huh7.5.1, and the expression of the transcript with threonine allele would not be up-regulated by HCV infection in an allele-specific manner. The missense mutation, as well as the overall elevation of gene expression, may have increased the replication efficiency of the HCV in Huh7 derivatives.

### Missense Mutation in RIG-I (T55I) Was Heterologous in the Huh7.5.1-8 Genome

**FIGURE 2:**
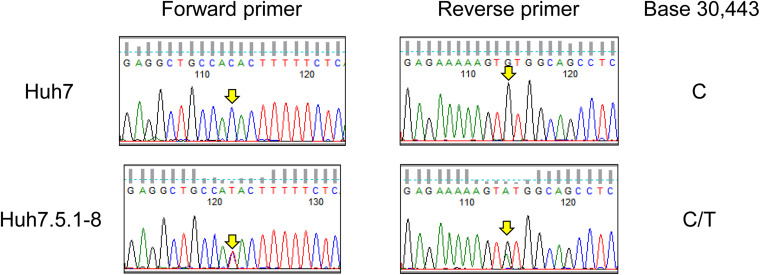
Sequence chromatograms of *DDX58* in Huh7 and Huh7.5.1-8. The genomic regions harboring the exon 2 of *DDX58* were amplified from Huh7 and Huh7.5.1-8 DNA samples using PCR and sequenced by the Sanger method. The arrows indicate a base 30,443 on NG_046918.1, and C-to-T transition at this position causes a T55I substitution of the DDX58 amino acid sequence.

As described in the above subsection, we found a T55I mutation in RIG-I that could contribute to the high permissiveness to HCV in Huh7.5.1-8. However, both genome sequencing and RNA-seq data showed the mutation is heterozygous in Huh7.5.1-8, contradicting previous findings that the mutation is homozygous in Huh7.5 (Sumpter et al., 2005). Therefore, we resequenced the genomic DNA from Huh7 and Huh7.5.1-8 using a Sanger sequencer and verified that the mutation is absent in Huh7 but heterozygous in Huh7.5.1-8 (Figure 2). Our recent study showed that a 55T allele transcript harbors an allele-specific large deletion and, presumably, non-functional in Huh7.5.1-8 (Saito et al., 2020). The results showed that, although T55I mutation is heterozygous in the Huh7.5.1-8 genome, all full-length RIG-I proteins have the T55I mutation.

In summary, the whole-genome sequencing of Huh7 and Huh7.5.1-8 provided various genetic characteristics of these cell lines, and some of them are, presumably, applicable to an in-house test for authentication of the Huh7 cell lineage. In addition, 53 genes were found to carry missense or LoF mutations specifically found in the Huh7 cell lineage. Among them, eight genes contained mutations observed only in Huh7.5.1-8 or mutations with higher frequency in Huh7.5.1-8. These mutations might be relevant to the phenotypic differences between Huh7 and Huh7.5.1-8. However, further studies will be needed to prove the hypothesis that one (or some) of the mutations is causative for the phenotypic differences among Huh7 cell sublines.

## Data Availability Statement

The sequences data were deposited in the DDBJ DRA database under the project ID PRJDB7928.

## Ethics Statement

Ethical review and approval was not required for the study on human participants in accordance with the local legislation and institutional requirements. Written informed consent for participation was not required for this study in accordance with the national legislation and the institutional requirements.

## Author Contributions

TE, MF, KH, and NO: study design. TY, KSai, and YS: performing experiments. MK, KSat, and NO: data analysis. MK, TY, KSai, KH, and NO: writing manuscript. All authors contributed to the article and approved the submitted version.

## Conflict of Interest

The authors declare that the research was conducted in the absence of any commercial or financial relationships that could be construed as a potential conflict of interest.

## Footnotes

1 http://iclac.org/wp-content/uploads/Cross-Contaminations-v8_0.pdf

2 https://github.com/J1011Ibb14029/kwmtools
