# Supplementary material for: Identification of Characteristic Genomic Markers in Human Hepatoma HuH-7 and Huh7.5.1-8 Cell Lines
Source: Front Genet. 2020 Oct 9;11:546106. doi: 10.3389/fgene.2020.546106 (PMC7581915; doi:10.3389/fgene.2020.546106)
Supplement: Supplementary file 2 [file Presentation_1.pdf]

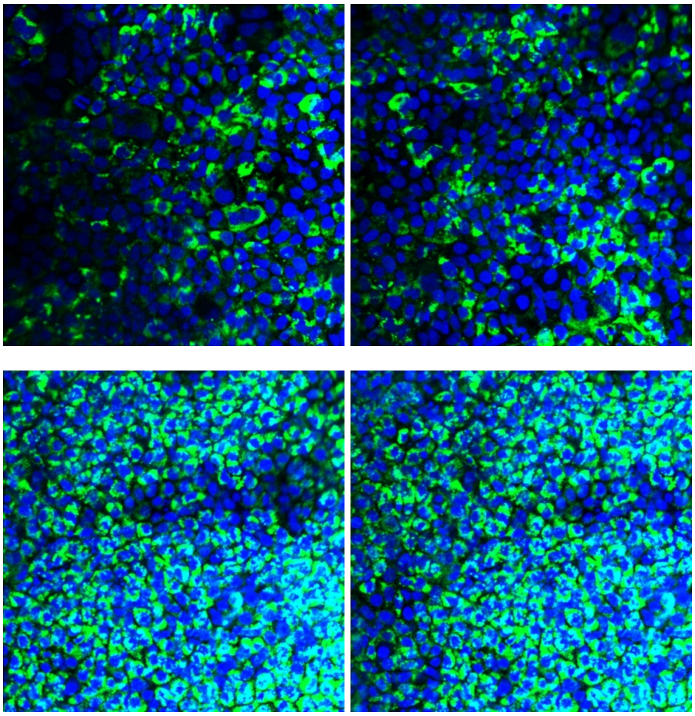

Figure S1. Fluorescent images of Huh7 and Huh7.5.1-8 cells after HCV infection

Huh7 (JCRB-0403) and Huh7.5.1-8 cells were incubated at 37ºC on a 24-well plastic plate with cover glasses (1 × $10^{5/}$well) with Dulbecco's modified Eagle medium supplemented with 10% (v/v) fetal bovine serum, 0.1 mM nonessential amino acids, 100 U/ml penicillin G, and 100 µg/ml streptomycin sulfate . After an overnight incubation, HCV (MOI = 1.0) was added to the culture and the cells were incubated at 37ºC for four days. After removing the culture supernatant, 1ml of 3.7% formaldehyde/PBS was added and the mixture was allowed to stand at room temperature for 30 mins. After fixing the cells, the cells were washed twice with 30 mM gycine/PBS, added with 0.2% Triton X-100/PBS 500 µl/well, and allowed to stand at room temperature for 10 mins. After permeabilization, the cells were washed twice with PBS, 5% skim-milk/PBS (500 µl/well) was added, and the mixture was allowed to stand at room temperature for 30 mins. Anti-HCV core mAb (2H9) was diluted to 1/500-fold with 5% skim-milk/PBS, and 50 µl was placed on parafilm, and cover glasses with the cell surface facing down was placed on the bottom and left at room temperature for 2 hours (Wakita et al. 2005). After returning the cover glasses to the 24-well plate, they were washed 3 times with PBS. A secondary antibody solution prepared by diluting Alexa Fluor 594 anti-mouse IgG (H+L) and DAPI 500 times with 5% skim-milk/PBS was treated in the same manner as the primary antibody and allowed to stand for 1 hour. The cells were washed three times with PBS. The cells were observed with a LSM700 microscopy. The blue and green fluorescent represent DAPI and secondary antibody for HCV core protein, respectively. For each sample, two images are shown.
